# Current Clinical Evidence on the Effect of General Anesthesia on Neurodevelopment in Children: An Updated Systematic Review with Meta-Regression

**DOI:** 10.1371/journal.pone.0085760

**Published:** 2014-01-20

**Authors:** Xin Wang, Zheng Xu, Chang-Hong Miao

**Affiliations:** Department of Anesthesiology, Shanghai Cancer Center and Cancer Institute, Shanghai Medical College, Fudan University, Shanghai, P.R. China; Iran University of Medical Sciences, Iran (Republic of Islamic)

## Abstract

**Background:**

Several epidemiological studies have been conducted to address the later effect of anesthesia on neurodevelopment in children. However, the results are still inconclusive.

**Methods:**

We here conducted a systematic review and meta-analysis to summarize the currently available clinical and epidemiologic evidence on the association of anesthesia/surgery with neurodevelopmental outcomes in children by searching PubMed, EMBASE, and Web of Science database (from January-1 2000 to February-1, 2013). The evaluation of neurodevelopment includes language and learning disabilities, cognition, behavioral development, and academic performance. Both retrospective and prospective studies were included. Data were abstracted from seven eligible studies. We estimated the synthesized hazard ratios (HR) and 95% confidence interval (CI) according to inter-study heterogeneity.

**Results:**

The pooled HR for the association of anesthesia/surgery with an adverse behavioral or developmental outcome was 1.25 (95% CI, 1.13–1.38, P<0.001; random-effects model) in children undergoing the first anesthesia before the age of 4-year. Then we analyzed the factors for this association using meta-regression method. It showed that it was the number of times of exposure (HR = 1.75, 95% CI 1.31–2.33; P<0.001) rather than the time at exposure before 4-year (HR = 1.08, 95% CI 0.87–1.34 for the effect of per 1-year early exposure; P = 0.47) is a risk factor for neurodevelopmental impairment.

**Conclusion:**

The current clinical evidence suggests modestly elevated risk of adverse neurodevelopmental outcomes in children who were exposed to anesthesia/surgery during early childhood, especially for those with multiple times of exposure. Due to limitation of retrospective studies, prospective investigations are needed to determine whether anesthesia/surgery is causative.

## Introduction

Although anesthesia in children is safe within short-term observation and has enabled children worldwide to undergo surgical procedure according to currently available clinical measurements, researchers are paying close attention to the question whether an administration of general anesthetic would lead to later neurodevelopmental (cognitive and behavioral) deficits in children.

An increasing volume of experimental and laboratory researches over the past decade have established a relationship between the administration of general anesthetics during periods of rapid brain growth and an increase in neuronal apoptosis and subsequent long-term neurodevelopmental impairment either in rodents or in nonhuman primates. [1]–[3] These results had raised the increasing concerns in clinicians and medical policy makers. A number, though not all, of case-control studies or subgroup analyses within cohort studies have found a potential association between the administration of anesthesia/surgery and subsequent cognitive or behavioral deficits. Moreover, DiMaggio et al have performed a Bayesian meta-analysis to investigate the association between pediatric anesthesia and neurodevelopmental impairment. [4] The synthesized hazard ratios (HRs) based on seven unadjusted and six adjusted measures for the association of anesthesia/surgery with an adverse developmental outcome were 1.9 (95% confidence interval [CI], 1.2–3.0) and 1.4 (95% CI, 0.9–2.2), respectively. However, they failed to include all eligible studies available at the time of analysis, e.g., the study by Hansen et al [5]. The author also included two ineligible papers by Guerra et al [6] and Roze et al [7], both of which studied the difference in neurological outcome between prolonged and short-term exposure to sedative and/or analgesic drugs rather than investigated the difference in neurological outcome between children exposed to anesthesia/surgery and those never exposed.

In the present study, we not only summarize the currently available clinical and epidemiologic evidence on the association of anesthesia/surgery with neurodevelopmental outcomes in children, but also answered whether early exposure is worse than late exposure, or whether multiple times of exposure is worse than a single exposure. Our findings may help to clarify the effect of anesthesia on neurodevelopmental outcomes. As well, doctors would have an idea of the proper number of times and the right timing of performance of anesthesia for children.

## Methods

### Study Identification and Data Extraction

**Figure 1:**
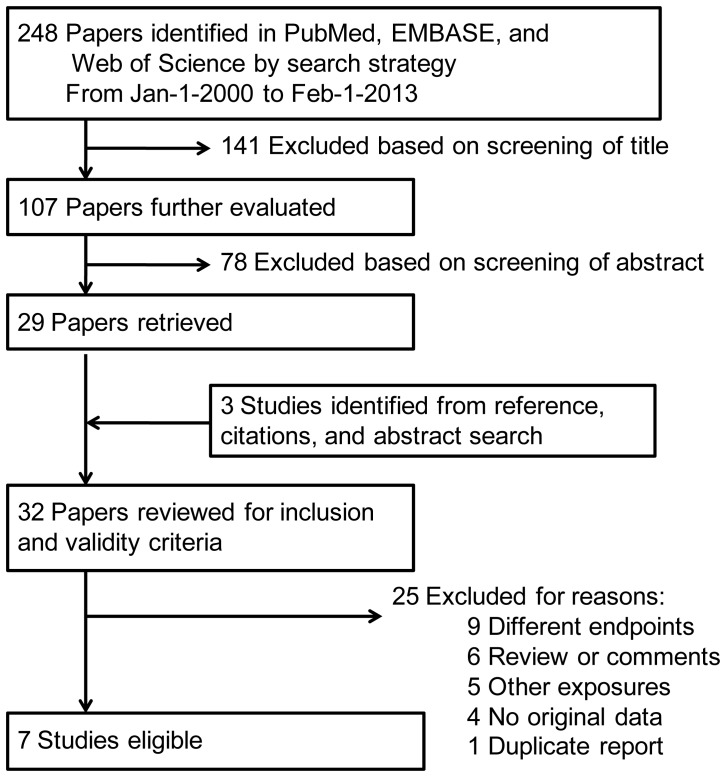
The literature search process.

We used PubMed, EMBASE, and Web of Science database (from January-1 2000 to February-1, 2013) to search relevant studies by using the following search terms: (anesthesia and [behavior* or development* or language or learning or neurodevelopment* or cognition or cognitive] and [pediatric or child*]). Only those published in English language were included; we did not define the minimum number of cases in studies to be included for meta-analysis. Either abstract or full text paper was eligible. We agree with DiMaggio et al that, to better reflect current clinical practice, only studies documented after 2000 years were eligible for inclusion. [4] The study flow chart is shown in **Figure 1**. By this search strategy, 248 papers were identified. After review their titles, 141 were excluded. All the left 107 were identified to be potentially eligible and their abstracts were read; if further possibly eligible, the full text papers were retrieved and read. Thirty-two papers were finally reviewed for inclusion criteria. The inclusion criteria were as following: (i) comparing the effect of general anesthesia on later neurodevelopment. The evaluation of neurodevelopment includes language and learning disabilities, cognition, behavioral development, and academic performance; (ii) independent retrospective or prospective study, and (iii) with sufficient available data to estimate HR with 95% CIs. Whenever available, we extracted the HR adjusted for other confounding factors, since the adjusted estimates might reflect the true odds of effect. After reviewing full text of all possibly eligible papers, seven eligible studies were selected for the present meta-analysis. [5], [8]–[14] The following variables were extracted from each study if available: first author’s surname, publication year, design type, total cases number, number of case in exposure, HR with 95% CIs of outcomes, time of exposure, timing at exposure, evaluation time, and evaluation items for neurodevelopment. The information was collected independently by two authors (W.X. and X.Z.), and any discrepancy were resolved by discussion. The study quality was assessed using the 9-star Newcastle-Ottawa Scale (The Newcastle-Ottawa Scale for assessing the quality of nonrandomized studies in meta-analyses. Ottawa, Canada: Dept of Epidemiology and Community Medicine, University of Ottawa. http://www.ohri.ca/programs/clinical_epidemiology/oxford.htm). We also followed the Preferred Reporting Items for Systematic Reviews and Meta-Analyses (PRISMA) statement for reporting systematic reviews that evaluate health care interventions [15].

### Meta-analysis and Statistical Methods

This systematic review and meta-analysis was planned, conducted, and reported in adherence to the standards of quality for reporting meta-analysis. [16] For each study, HR was retrieved to estimate the association between anesthesia and neurodevelopmental outcomes. The heterogeneity among studies was assessed by Cochran chi-square Q statistics and I-square statistics, which determined the appropriate use of either fixed-effects (Mantel-Haenszel method) or random-effects (DerSimonian and Laird method) model. Heterogeneity was considered as either a P-value <0.05 or I-square >25%. [17] The potential publication bias was assessed graphically in a funnel plot of ln[OR] against its standard error (SE), and the degree of asymmetry was tested using Egger’s test and Begg’s test. Influence analysis (sensitivity analysis) was conducted by omitting each study to find potential outliers.

We also computed the trend from the correlated ln(HR) estimates across categories of exposure levels (such as time at exposure [per 1-year early]) by meta-regression as previously described. [18], [19] Via meta-regression, we converted the effect of time at exposure to a regression coefficient and its standard error (SE) corresponding to a continuous representation per 1-year early exposure. The summary measures of HR per 1-year early exposure could be interpreted as the incidence rate ratio for the outcome with each 1-year early exposure. Similar procedure of meta-regression was performed to test the effect of number of times of exposure on outcome. We made above calculations assuming a log linear relationship between HR and early time exposure. A two-tailed P value of <0.05 was considered statistically significant. All of the statistical analyses were performed using Stata/SE version 10.0 (Stata Corporation, College Station, TX).

## Results

### Study Characteristics

There were seven studies [5], [8]–[14] addressing the issue of association of anesthesia/surgery with neurodevelopmental outcomes as shown in **Table 1**. A total of 44,143 children were included and 5,546 of them had experienced general anesthesia due to surgery procedures. The minimum age at exposure was at birth to six months and the maximum age was at the age of four years, with a mean age of three years. Year of birth ranged from 1976 to 2005. Children after exposure were followed up from one year to 16 years.

**Table 1:** Characteristics of eligible studies for meta-analysis.

Author	Year	Design Type	StudyQuality§	Country	Exposed/Total (n)	HR	95% CI	Number of Timesof Exposure	Time at Exposure(m)	EvaluationTime	Birth (y)	Evaluation Items
Bartels [8]	2009	PB, prospective case-control	7	Netherlands	1,078/2,286	1.7	0.6–5	ever	<36	near age 12	1986–1995	Educational achievementand cognition
DiMaggio [9]	2009	PB, retrospective cohort	8	USA	383/5,433	2.3	1.3–4.1	ever	<36	before age 4	1999–2002	Developmental or behavioraldisorder
Kalkman [10]	2009	Clinical, retrospectivecase-control	6	Netherlands	178/243	1.38	0.59–3.22	ever	<6	Medianage 14.5	1981–1995	Behavioraldevelopment
						1.19	0.45–3.18	ever	6–12			
						1.2	0.45–3.2	ever	12–24			
Wilder [11], [12]	2009,2011	PB, retrospective cohort	8	USA	593/5,357	1.2	0.99–1.46	ever	<48	at age 5	1976–1982	Learningdisabilities
						1.00	0.79–1.27	1	<48			
						1.59	1.06–2.37	2	<48			
						2.60	1.60–4.24	≥3	<48			
						1.06	0.77–1.46	1*	<24			
						2.12	1.26–3.54	≥2*	<24			
DiMaggio [13]	2011	PB, retrospective cohort	8	USA	304/10,450	1.6	1.4–2.8	ever	<36	before age 6	1999–2005	Developmental andbehavioral disorders
						1.1	0.8–1.4	1	<36			
						2.8	2.5–3.1	2	<36			
						4.0	3.5–4.5	≥3	<36			
Hansen [5]	2011	PB, prospective cohort	9	Denmark	2,689/17,263	1.14	1–1.31	ever	<36**	at age15–16	1986–1990	Academicperformance
Ing [14]	2012	PB, prospective cohort	8	Australia	321/2,868	1.69	1.13–2.53	ever	<36	at age 10	1989–1992	Cognition
						1.73	1.04–2.88	1	<36			
						1.92	0.81–4.55	≥2	<36			

CI, confidence interval; HR, hazard ratio; PB, Population-based.

data from paper by Flick et al using the same study population but with different cut-off of timing at exposure.

during infancy (probably within 36 months).

evaluated by the 9-star Newcastle-Ottawa Scale.

Of the seven study findings, one reported the unadjusted associations of exposure to anesthetic agents with neurodevelopmental outcome. Although we did not find the HR and 95% CI in the original paper, DiMaggio et al had calculated them in their previous meta-analysis. [4] The findings from other six papers had adjusted HRs for association between exposure to anesthesia and neurodevelopmental outcomes. Five of the seven studies reported positive associations either in the overall or in the subgroup analyses. For one study which was reported by two papers, [11], [12] we used the data from the paper with the maximum sample size; [11] of note, these two papers used different cut-off values of time at exposure (one was at age of four years and the other was two). The quality of studies evaluated by the Newcastle-Ottawa Scale is shown in **Table 1**.

### Effect Measure Synthesis

**Figure 2:**
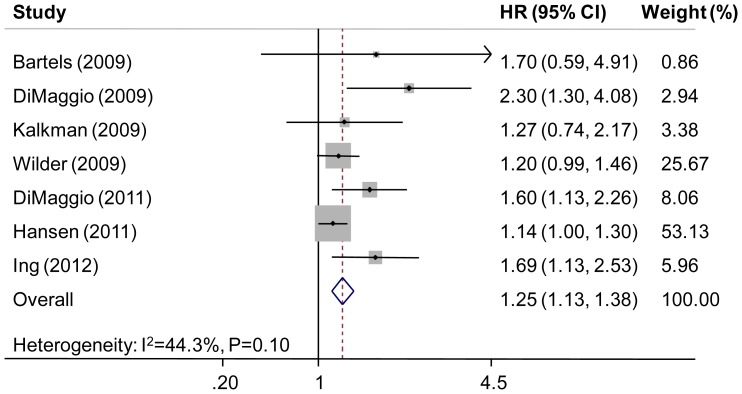
Individual study and overall hazard ratios of relationships between general anesthesia and neurodevelopmental impairment in children. The size of each square is proportional to the weight of the study. For the combined result, the length of the diamond represents the 95% CI of the summary.

**Figure 3:**
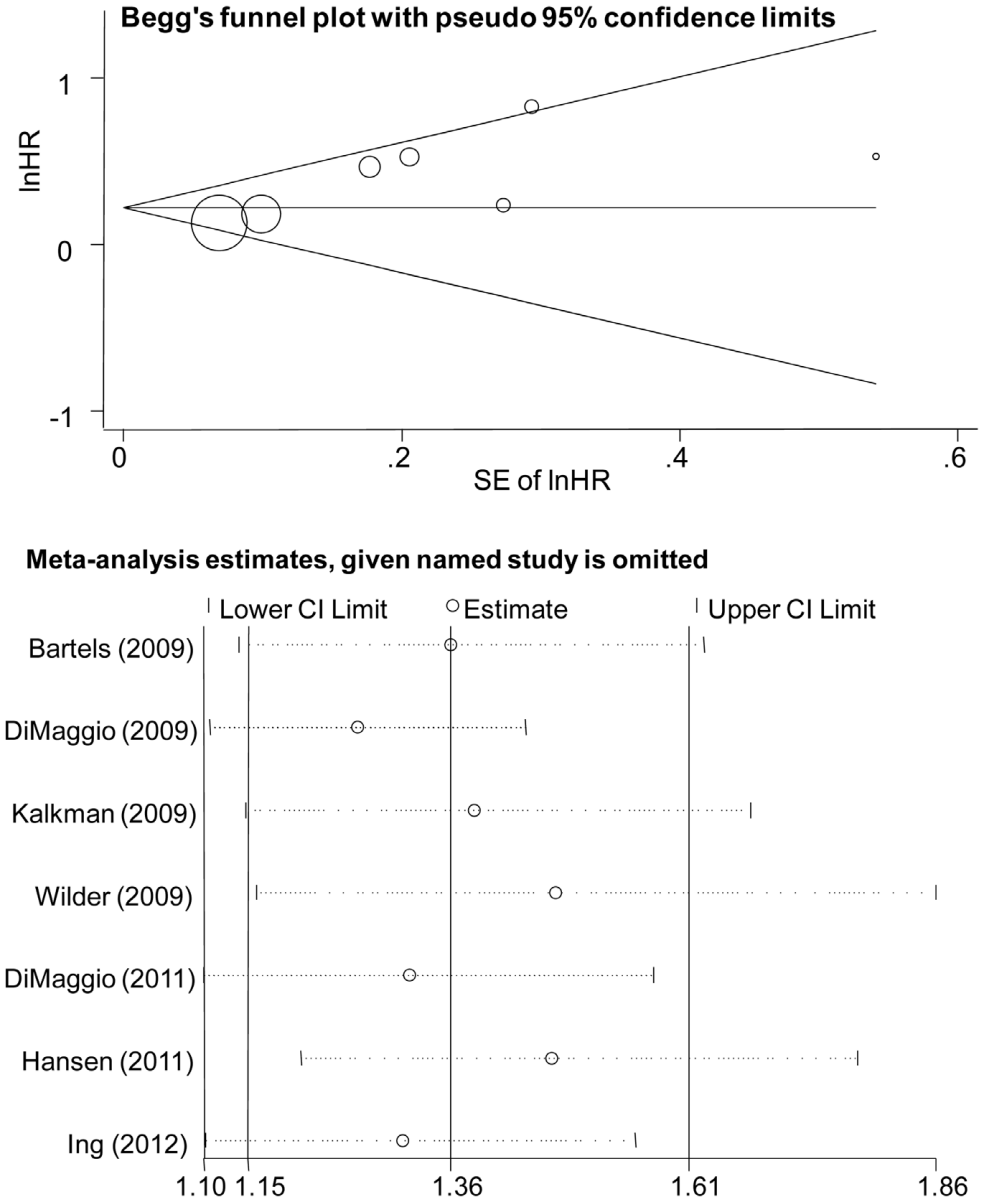
Publication bias and influence analysis of meta-analysis. A. Funnel plots of studies included in the meta-analysis. The vertical axis represents ln[HR] and the horizontal axis means the standard error of ln[HR]. Horizontal line and sloping lines in funnel plot represent summary HR and expected 95% CIs for a given standard error, respectively. Area of each circle represents contribution of the study to the pooled OR. B. Influence of individual studies on the pooled HR. The vertical axis indicates the overall HR and the two vertical axes indicate its 95% CI. Every hollow round indicates the pooled OR when the left study is omitted in this meta-analysis. The two ends of every broken line represent the respective 95% CI.

The Q statistic for the seven estimates for any exposure was 10.72 (P = 0.10) with an I-square of 44.3%. Therefore, the random-effects model was used to analyze the data and an association between anesthesia/surgery with neurodevelopmental impairment was observed with a pooled HR of 1.25 (95% CI, 1.13–1.38, P<0.001; **Figure 2**). Moreover, either graphical inspection for funnel plots (**Figure 3A**) or quantitative evaluation from Egger’s test and Begg’s test indicated an absence of publication bias (P = 0.133 and P = 0.099, respectively). Influence analysis was further conducted to ascertain the influence of each study on the overall HR (**Figure 3B**). No individual study affected the overall OR dominantly, because omission of any single study made no substantial difference.

### Meta-regression Analysis

**Figure 4:**
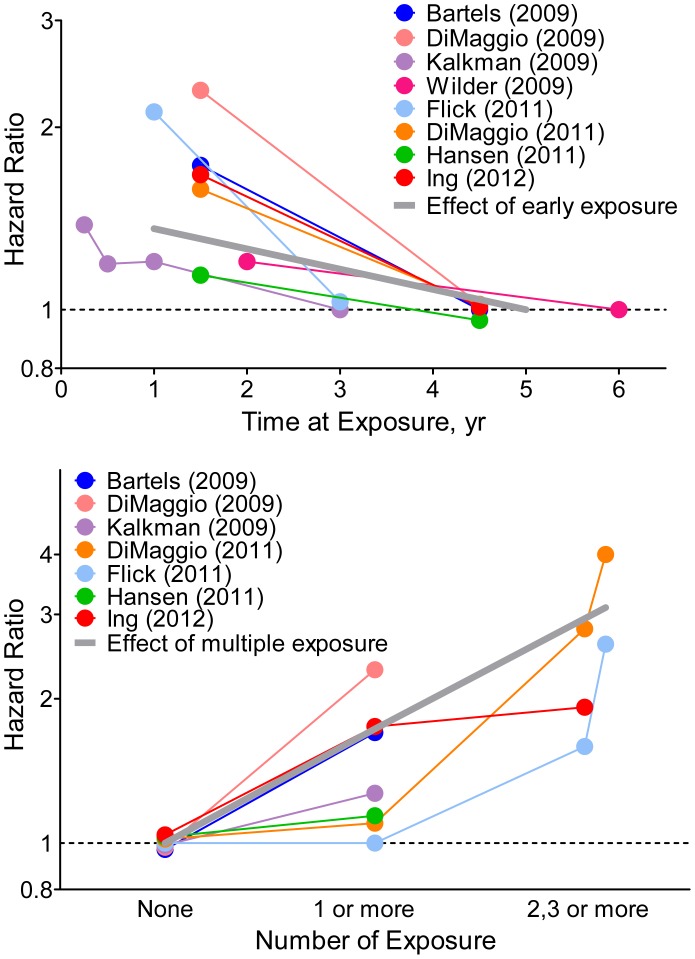
Effect of time at exposure and number of times of exposure of general anesthesia on neurodevelopment in children. A. Relationship between categories of time at exposure and hazard ratio (HR) of later neurodevelopmental deficits in each analytical group. The line for each individual study is located over the range of time at exposure. The gray thick line indicates the effect of time at exposure (per 1-year early) on neurodevelopmental deficits using a meta-regression analysis. The slope of gray line represents the change in the log HR per 1-year early. The vertical axis is on a log scale. The result of effect of number of times of exposure on neurodevelopmental deficits is shown in B. The gray thick line indicates the effect of number of times of exposure on neurodevelopmental deficits.

Now that we have demonstrated an association between anesthesia/surgery and neurodevelopmental impairment, a meta-regression was further performed to explore whether the risk of neurodevelopmental impairment could be predicted according to two specific parameters, i.e., time at exposure and number of times of exposure. The HR results of individual eligible studies listed in **Table 1** are plotted in **Figure 4**, which shows the HRs for categorical representations of time at exposure (**Figure 4A**) or number of times of exposure (**Figure 4B**). For the time at exposure, the slope of the gray bold line (by log converted HR) in **Figure 4A** represented the risk of neurodevelopmental impairment due to per 1-year early exposure to anesthesia/surgery. The HR of effect of early exposure per year is 1.08, with 95% CI of 0.87–1.34 and a not significant P value of 0.47, indicating time at exposure (before age of 4 years) might has limited effect on neurodevelopmental impairment. However, physician should take note that children with early exposure may take a higher risk of neurodevelopmental impairment compared with those with later exposure (8% increased risk per 1-year early and 17% per 2-year early, though with no statistical significance). For the number of times of exposure, we classified the exposure times into three categories, never, once or ever, and twice and more according to the original data. The hazard of multiple exposure was about 1.75 (95% CI, 1.31–2.33) with a significant P value of <0.001, indicating the number of times of exposure is important for neurodevelopmental impairment, and multiple exposures to anesthesia/surgery before the age of 4 years should be avoided.

## Discussion

Results of the present meta-analysis suggest a potential influence of anesthesia/surgery on the later neurodevelopmental (cognitive and behavioral) deficits. Moreover, it seems that the number of times of exposure rather than the time at exposure before 4 years of age is a more significant risk factor for neurodevelopmental impairment.

Our findings from pooled meta-analysis are consistent with most, although not all, findings from currently available literatures. A number of behavioral and psychiatric sequelae late in life might be caused by health care interventions in natal or infant period. [20] The results of such investigations present us with the paradox that interventions known to be beneficial for infants and children may have unexpected adverse consequences in adulthood. Anesthetic agents themselves might be associated with adverse outcomes. Hattori et al have investigated the effect of anesthetic agents on the developmental disorders of children in an indirect way by investigating the children having undergone general anesthetic delivery. In a case series comparing 11,939 births in a hospital where routinely administered anesthetic agents were administered during delivery with 19,580 children in three hospitals where non-anesthetic deliveries were common, there were 0.2% cases of autistic disorder in the anesthetic-exposed group compared to 0.09% cases of autistic disorder in the unexposed group, with a statistically significant difference. [21] In contrast, our meta-analysis pooled the pieces of direct epidemiological evidence in which the children were directly under the exposure of anesthesia. Our results might provide some useful information for pediatrics as well as for health policy makers. First, a procedure of anesthesia/surgery for children younger than 4 years is probably associated with later impairment in neurodevelopment. Second, this positive association is influenced by the number of times of exposure to anesthesia/surgery but not timing of exposure, indicating that an early surgery in children is probably acceptable and doctors should try to avoid multiple times of anesthesia/surgery procedures in children. Early evidence also suggest that in a single procedure of surgery, dose of anesthetic agents and time of anesthesia might have very limited influence on the neurodevelopmental outcomes [6], [7].

Some limitations should be declared. First, our meta-analysis is limited by the nonrandomized and retrospective nature of the included studies. Second, there should be other prognostic factors not controlled in the meta-analysis. Differences in different surgical techniques, varying patient populations, changes in definition of recurrence, and difficulty with long-term follow-up, all hamper firm conclusions. Third, we only included studies published in English language and would introduce so called “English language bias” that may reduce the precision of combined estimates of treatment effects; this problem however exists in most currently published meta-analysis and systematic review. Forth, pediatric anesthetic neurotoxicity is a complicated and complex issue. There are many variables at play in addition to the potentially toxic effects of anesthesia, including maternal health, drug exposures during pregnancy and delivery, preexisting medical conditions in the child, and environmental or ecological characteristics. As well, the evaluation of neurodevelopment should use standard measurement forms, and different forms would results in different outcomes of neurodevelopmental assessment. Given this complexity, observational studies are hard put to demonstrate unequivocal associations or risk. The results of this meta-analysis must be taken cautiously within the context of the data upon which it is based. After all, no statistical approach is a panacea for potentially biased or confounded data.

In conclusion, we have found that a procedure of anesthesia/surgery before 4 years old is possibly associated with later neurodevelopmental deficits. Multiple times of exposure to anesthesia should be avoided. However, due to the limitation of retrospective studies, prospective studies are needed to determine whether the association between anesthesia/surgery and neurodevelopmental deficits is causative.

## Supporting Information

Checklist S1 The PRISMA checklist.
(DOC)
